# Postoperative rectovaginal fistula: stoma may not be necessary—a French retrospective cohort

**DOI:** 10.1007/s10151-024-03013-2

**Published:** 2024-10-03

**Authors:** Maëlig Poitevin, Jean-Francois Hamel, Marie Ngoma, Charlène Brochard, Emilie Duchalais, Laurent Siproudhis, Jean-Luc Faucheron, Vincent de Parades, Arnaud Alves, Eddy Cotte, Mehdi Ouaissi, Valérie Bridoux, Lisa Corbière, Pablo Ortega-Deballon, Fawaz Abo-Alhassan, Bertrand Trilling, Aurélien Venara

**Affiliations:** 1 Department of Medicine, University of Health, Angers, France; 2 Department of Visceral Surgery, CHU Angers (Angers University Hospital), University of Angers, 9 Angers, Cedex France; 3 Department of Biostatistics, La Maison de La Recherche, University Hospital of Angers, 9 Angers, Cedex France; 4 Institut Léopold Bellan, Groupe Hospitalier Paris Saint-Joseph, Service de Proctologie Médico-Chirurgicale, Paris, France; 5 Unité D’explorations Fonctionnelles Digestives, CHU Rennes Pontchaillou, Rennes, France; 6 Unité de Proctologie, CHU Rennes Pontchaillou, Service Des Maladies de L’appareil Digestif, Rennes, France; 7 Department of Digestive Surgery, University Hospital of Nantes, Nantes, France; 8 UMR 5525, Univ. Grenoble Alpes, CNRS, Grenoble INP, CHU Grenoble Alpes, TIMC, VetAgro Sup, 38000 Grenoble, France; 9 Colorectal Surgery Unit, Visceral Surgery and Acute Care Surgery Department, Grenoble Alps University Hospital, Grenoble, France; 10 Department of Digestive Surgery, University Hospital of Caen, Caen Cedex, France; 11 UMR INSERM U1086 Anticipe, Centre François Baclesse, Caen, Cedex France; 12 Department of Digestive Surgery, Hôpital Lyon Sud, CHU Lyon, Cedex, France; 13 Faculty of Medicine of Lyon Sud-Charles Mérieux, University Lyon 1, Cedex, France; 14 Department of Digestive, Oncological, Endocrine, Hepato-Biliary Pancreatic and Liver Transplant Surgery, Trousseau Hospital, Chambray Les Tours, France; 15 Department of Digestive Surgery, Rouen University Hospital, Rouen, France; 16 Department of Digestive Surgery, CHU Rennes Pontchaillou, Rennes, France; 17 Department of Digestive Surgery, Dijon University Hospital, Dijon, France; 18 SFR ICAT, CHU Angers, HIFIH, University of Angers, 9 Angers, Cedex France

**Keywords:** Rectovaginal fistula, Postoperative, Stoma, Iatrogenic, Surgery

## Abstract

**Background:**

Postoperative rectovaginal fistula leads to a loss of patients’ quality of life and presents significant challenges to the surgeon. The literature focusing specifically on postoperative rectovaginal fistulas is limited. The objective of the present study is to identify factors that can enhance the success of the management of this postoperative rectovaginal fistula.

**Methods:**

This retrospective multicentric study included all patients undergoing surgery for rectovaginal fistulas, excluding those for whom the etiology of rectovaginal fistula was not postoperative. The major outcome measure was the success of the procedure.

**Results:**

A total of 82 patients with postsurgical fistulas were identified, of whom 70 were successfully treated, giving a success rate of 85.4%. On average, these patients required 3.04 ± 2.72 interventions. The creation of a diversion stoma did not increase the success rate of management [odds ratio (OR) = 0.488; 95% confidence interval (CI) 0.107–2.220]. Among the 217 procedures performed, 69 were successful, accounting for a 31.8% success rate. The number of interventions and the creation of a diversion stoma did not correlate with the success of management. However, direct coloanal anastomosis was significantly associated with success (OR = 35.06; 95% CI 1.271–997.603; *p* = 0.036) as compared with endorectal advancement flap (ERAF). Other procedures such as Martius flap did not show a significantly higher success rate.

**Conclusion:**

The creation of a diversion stoma is not necessary in closing a fistula. ERAF should be considered as a first-line treatment prior to proposing more invasive approach such as direct coloanal anastomosis.

## What does the study add to the literature?

**Table:** 

Mini-invasive surgery such as endorectal advancement flap should be proposed as the first approach to treat postoperative rectovaginal fistula, with a success rate of near 30% prior proposing a more invasive surgery (coloanal or colorectal anastomosis). Diverting stoma is not mandatory in such situation.	

## Introduction

Rectovaginal fistula (RVF) is defined as an abnormal communication between the rectum and the vagina through the rectovaginal septum. It is characterized by the passage of rectal contents (gas and/or feces) into the vagina. Rectovaginal fistulas account for approximately 5% of all anorectal fistulas, which have an incidence in Europe ranging from 1.20 to 2.80 per 10,000 people each year [1, 2]. Acquired rectovaginal fistulas have numerous etiologies, including inflammatory, obstetrical, and postoperative causes [3, 4]. These fistulas significantly impact the quality of life (social, sports, etc.) of affected patients and lead to a deterioration in their emotional well-being [5, 6].

To the best of our knowledge, there are no specific recommendations for the surgical management of RVF. Indeed, research published to date largely focuses on the management of rectovaginal fistulas without considering their etiology, even though it has been demonstrated that the etiology of fistulas plays an important role in determining the success rate of fistula closures [7, 8]. Moreover, although the literature on postoperative rectovaginal fistulas is scarce, their incidence ranges from 0.58% to 5.1% depending on the performed intervention [9, 10].

Numerous surgical techniques have described, all with varying degrees of success. These include endoscopic techniques, more or less invasive surgical procedures involving the use of flaps (vaginal, mesorectal, peritoneal, muscular), autologous buccal mucosa grafts, or platelet-rich plasma [3, 11–22]. To date, no technique has succeeded in establishing itself as the standard treatment for postoperative rectovaginal fistulas. The lack of standardization in treatment is even more problematic because the repair of rectovaginal fistulas resulted in complications in 13.2% of cases, with 7.9% of all complications being serious [23].

The aim of this study was to provide new insights into postoperative rectovaginal fistulas and their management.

## Materials and methods

We conducted a retrospective multicenter cohort study including all patients referred for surgical treatment for postoperative rectovaginal fistulas according to a database performed across 10 centers for all the patients undergoing surgery for RVF. A previous study using this database only included patients whose fistula was secondary to an obstetrical anal sphincter injury (OASI) [24].

To be included in the present analysis, the patients needed to have undergone surgery for RVF secondary to proctological, gynecological, or colorectal surgery between 2005 and 2020. Patients were excluded if the fistula was not secondary to a surgical procedure [e.g., RVF due to AOSI, traumatic RVF and RVF due to Crohn’s disease (CD)] if no surgical procedure had been performed to close the fistula, if a terminal or diverting stoma had not been created without a repair procedure, or if the patient had expressed his or her opposition to participating in retrospective studies. If patients with CD experienced a RVF after any surgery for any other cause, they could be included in this study.

The study received the approval of our local ethics committee (2021/034) and the database was declared to the Commission National Informatique et Libertés (France’s national data protection agency) (ar21-0014v0).

Patients were selected according to the reason why they had been admitted to hospital^25^. The following data were collected in an anonymized database:

- Demographic data: age, body mass index (BMI), medical history;
- Surgical procedure that led to the fistula;
- For each procedure performed to cure the fistula:
  o Type of surgery performed;
  o Creation of a diverting stoma;
  o Efficacy of the surgery;
- Follow-up after the last surgery.

The main outcome measure was the success of the surgery, as clinically defined in the previous study, as the absence of stool or gas in the vagina [24]. If the patient had undergone a definitive stoma procedure to treat the suppuration, the management was not considered to have been successful.

### Management of the patient

There was no standard perioperative management of the patients across the different centers [24]. All the centers involved were university hospitals that reported at least 30 cases of RVF each during the study period. The procedures and the order in which they were performed were left at the surgeon’s discretion. Sphincter-sparing procedures were primarily favored as recommended.

### Statistical analysis

Continuous covariates were described using mean and standard deviations and compared using student tests, whereas categorical covariates were described using percentages and compared using chi-squared tests.

The relationship between the efficacy of surgery and the factors considered to be associated with it (e.g., diverting stoma, type of procedure, number of recurrences prior to the considered surgical treatment, BMI, and age of the patient) was assessed through a mixed logistic model considering patients as a random effect covariate. This enabled us to take into account the possible recurrences of the rectovaginal fistula among given patients.

In this model, the variance–covariance structure was defined as unstructured.

All statistical analyses were performed assuming a type I error probability of 0.05.

## Results

**Fig. 1:**
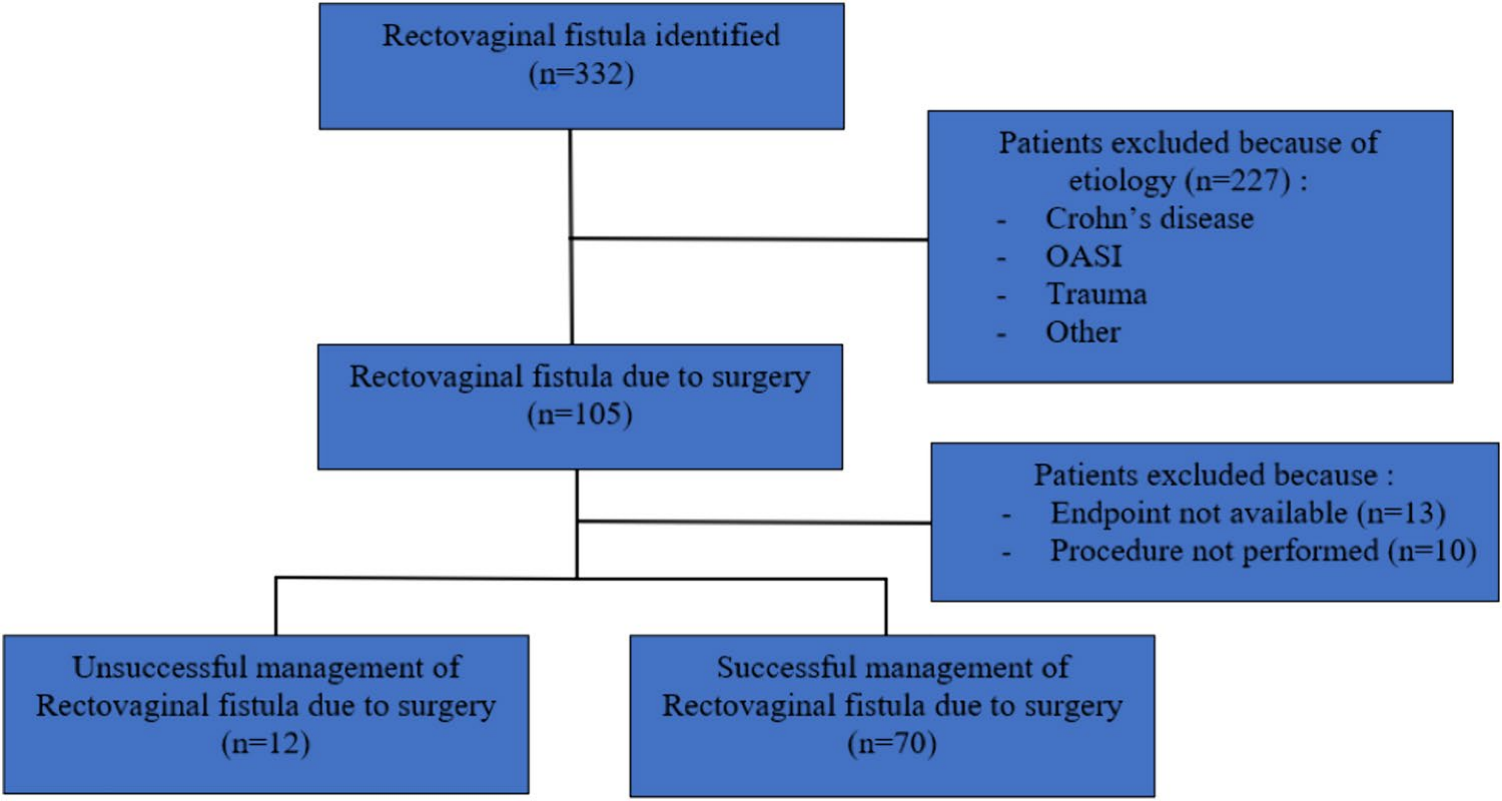
Flow chart of our population

A total of 332 patients with rectovaginal fistulas (RVF) were identified. Postoperative rectovaginal fistula was found in 105 (31.6%) (Fig. 1). Twenty-three patients were excluded because the primary endpoint was not available (*n* = 13) or because no repair procedure was performed (*n* = 10).

In the end, 82 patients with treated postoperative RVF were considered in this study. The mean age of these 82 patients was 48 years (± 17) and the mean BMI was 24.1 kg/m^2^ (± 5.1).

Among the 82 patients, a mean of 3 procedures (± 2.7) was performed per patient with no significant difference between the successful and unsuccessful groups (*p* = 0.08). A diverting stoma was performed in 60 patients (73.2%) during the course of the management. After the last intervention collected, 70 patients had a successful management of RVF (85.4%) (Table 1).

**Table 1:** Comparison of patient characteristics between the group with an unsuccessful management and the group with a successful management

	Overall population (82)	Unsuccessful (*n* = 12; 14.6%)	Successful (*n* = 70; 85.4%)	*p*-value
Mean age, years	48.26 ± 17.36	50.28 ± 17.71	47.92 ± 17.40	0.66
BMI, kg/m^2^	24.05 ± 5.06	24.14 ± 3.95	24.04 ± 5.26	0.96
Proctologic medical history	18 (21.95%)	4 (33.33%)	14 (20.00%)	0.30
Crohn’s disease medical history	1 (2.00%)	1 (10.00%)	0 (0.00%)	0.04
Parity	1.39 ± 1.61	2.40 ± 1.82	1.23 ± 1.54	0.13
Diverting stoma	60 (73.17%)	10 (83.33%)	50 (71.43%)	0.39
Mean rate of surgical procedures performed with a concomitant diverting stoma per patient	0.63 ± 0.44	0.63 ± 0.43	0.63 ± 0.44	0.95
Mean number of procedures performed/patient	2.65 ± 2.55	3.50 ± 2.65	2.50 ± 2.53	0.21
Mean number of procedures performed with a diverting stoma/ patients	1.66 + −2.10	1.67 ± 1.07	1.66 ± 2.24	0.99
Rectal cancer medical history	19 (23.17%)	4 (33.33%)	15 (21.43%)	0.37
Radiotherapy medical history	15 (18.29%)	5 (41.67%)	10 (14.29%)	0.02
Initial surgery				0.12
Gynecological surgeries (benign/ endometriosis)	19 (27.94%)	3 (27.27%)	16 (28.07%)	
Anterior rectal resections	36 (52.94%)	4 (36.36%)	32 (56.14%)	
Surgeries for gynecological cancers	1 (1.47%)	1 (9.09%)	0 (0.00%)	
Surgery for rectocolitis	6 (8.82%)	1 (9.09%)	5 (8.77%)	
Proctological surgeries or TAMIS	6 (8.82%)	2 (18.18%)	4 (7.02%)	

At baseline, there was no significant difference between the successful and unsuccessful groups of patients in terms of age, BMI, proctological history, parity, rectal cancer, and diverting stoma (Table 1). Patients were more likely to have a history of radiotherapy and/or Crohn's disease in the unsuccessful group (*p* = 0.02 and *p* = 0.04, respectively) (Table 1). In the cohort, 60 patients had a diverting stoma during the management (*p* = 0.39) ((Table 1). The initial surgeries performed were grouped into five categories, including gynecological surgeries, anterior rectal resections, surgeries for gynecological cancers, surgeries for rectocolitis (total coloproctecomy with ileoanal anastomosis), and proctological surgeries.

Description of surgery cohort

Out of the 82 patients, 217 interventions were performed, of which 69 procedures were successful (31.9%) and 137 (63.1%) were associated with the creation of a diverting stoma (Table 2). The presence of a diverting stoma was comparable in both the success and non-success arms (*p* = 0.66). The most frequently performed procedure was the endorectal advancement flap (44/217, i.e., 22.68% of all procedures) followed by the placement of a seton (22/117, 11.3%), and vaginal suture (22/217, 11.3%) (Table 2). More invasive procedures were performed in 8/217 (coloanal pull-through procedure), 5/217 (Soave procedure), 6/217 (direct coloanal anastomosis), and 11/217 cases (colorectal anastomosis).

**Table 2:** Comparison of the characteristics of the procedures according to the successful of unsuccessful characteristic of the procedure

	Overall procedures (*n* = 217)	Unsuccessful (*n* = 148)	Successful (*n* = 69)	*p*-value
Diverting stoma	137 (63.13%)	92 (62.16%)	45 (65.22%)	0.66
Surgical procedure				0.001
Seton	22 (11.34%)	18 (14.40%)	4 (5.80%)	
Glue	11 (5.67%)	10 (8.00%)	1 (1.45%)	
Endorectal advancement flap	44 (22.68%)	30 (24.00%)	14 (20.29%)	
Martius flap	9 (4.64%)	9 (7.20%)	0 (0.00%)	
Gracilis flap	4 (2.06%)	3 (2.40%)	1 (1.45%)	
Ovesco clip	11 (5.67%)	7 (5.60%)	4 (5.80%)	
Vaginal suture	22 (11.34%)	12 (9.60%)	10 (14.49%)	
Plug	12 (6.19%)	9 (7.20%)	3 (4.35%)	
Musset procedure	3 (1.55%)	1 (0.80%)	2 (2.90%)	
Beaulieu procedure	8 (4.12%)	5 (4.00%)	3 (4.35%)	
Soave	5 (2.58%)	3 (2.40%)	2 (2.90%)	
Coloanal anastomosis	6 (3.09%)	1 (0.80%)	5 (7.25%)	
Anterior resection of rectum	11 (5.67%)	1 (0.80%)	10 (14.49%)	
Other	26 (13.40%)	16 (12.80%)	10 (14.49%)	
Number of procedures	3.04 ± 2.72	3.26 ± 2.72	2.57 ± 2.67	0.08

Multivariate analysis of predictive factors of success.

In a multivariate analysis (Table 3), the creation of a diverting stoma did not increase the success rate of the overall management (OR = 0.71; 95% CI 0.12–4.29), as well as the number of interventions did not (OR = 0.90; 95% CI 0.71–1.16).

**Table 3:** Multivariate analysis of the predictive factors for successful management

	Odds ratio	95% confidence interval	*p* value
Diverting stoma	0.71	0.12–4.29	0.71
Number of procedures	0.90	0.71–1.16	0.42
Age > 60 years	0.49	0.08–2.81	0.42
BMI > 25 Kg/m^2^	0.76	0.07–8.28	0.82

In another model of multivariate analysis aiming to assess the success of the procedure (Table 4), the creation of a diversion stoma did not increase the success rate of the success of the surgery (OR = 0.49; 95% CI 0.11–2.22), just as the number of surgical procedures performed had no effect on the success rate (OR = 1.09; 95% CI 0.81–1.48). Regarding the various surgical techniques employed, only direct coloanal or colorectal anastomosis showed a significant increase in the success of management as compared with the ERAF (OR = 35.60; 95% CI 1.27–997.60; *p* = 0.04).

**Table 4:** Multivariate analysis of the predictive factors for successful surgery

	Odds ratio	95% confidence interval	*p* value
Diverting stoma	0.49	0.11–2.22	0.35
Surgical procedure			
Seton	0.11	0.005–2.48	0.17
Glue	0.05	0.001–2.67	0.14
Gracilis flap	0.92	0.19–4.54	0.92
Vaginal suture	1.95	0.24–15.81	0.53
Musset procedure	1.96	0.02–202.75	0.78
Beaulieu procedure	1.42	0.14–14.65	0.77
Coloanal procedure	35.6	1.27–997.60	0.04
Number of procedures	1.09	0.81–1.48	0.56
BMI > 25 kg/m^2^	2.87	0.33–24.77	0.34
Age > 60 years	2.72	0.54–13.63	0.22

## Discussion

Out of the 82 patients with post-surgical RVF in our cohort, 70 were successfully treated, giving an overall success rate of 85.4%. On average, these patients required 3.04 ± 2.72 interventions. The creation of a diversion stoma did not increase the success rate of management (OR = 0.49; 95% CI 0.11–2.22). Among the 217 procedures performed, 69 were successful (31.8%). Performing a direct coloanal or colorectal anastomosis was significantly associated with success as compared with ERAF (OR = 35.06; 95% CI 1.27–997.60; *p* = 0.04). Other procedures did not show a significantly higher success rate.

Quite like in our cohort, the literature reports that RVF due to postoperative complications accounts for 6.3–40.2% of all cases of RVF [7, 25, 26]. Our study also reported that the surgical procedures that had caused RVF were predominantly anterior rectal resections (52.94%) and gynecological surgeries (27.94%). This is in line with the findings reported in the literature, notably by Drefs et al., who reported these procedures as caused RVF in 59.5% and 27.0% of cases, respectively [7]. Moreover, to the best of our knowledge, our cohort is the largest cohort of postoperative rectovaginal fistulas.

Our study found that creating a diversion stoma had no benefits with regard to improving the chances of fistula closure. There is no real consensus in literature on this point. Barugola et al. [27] found a higher success rate in the diversion stoma group (*p* value = 0.003) in a study of a cohort of 37 postoperative fistulas. Furthermore, Corte et al. [28] found an odds ratio of 3.5 (1.4–8.7) in favor of using a diversion stoma in a cohort of 79 RVF (including only 25 postoperative RVF). Then again, studies such as Fu et al. [29] and Lambertz et al. [30] found that creating a diversion stoma brought no benefit for patients, with respective *p* values of 0.490 and 0.603 and sample sizes of 63 and 62 RVF, including 18 and 15 postoperative ones. Abo-Alhassan F. et al. [31] found that stoma was associated with success rate in a cohort including multi-etiology RVF. Our study has the strength that it included only patients with postoperative RVF while all of the previous studies combined various etiologies of rectovaginal fistulas, irrespective of the fact that the success rate differs according to the etiology of the RVF. However, due to the retrospective nature of our study, it is important to consider that diversion stomas were probably more commonly performed in the most severe cases, thus potentially diminishing the effectiveness of diversion stomas and possibly representing a bias. Regarding the question of creating diversion stomas for postoperative rectovaginal fistulas, therefore, we agree with the conclusions of the German S3-Guidelines, recommended by Ommer et al. [32]: these state that creating diversion stomas should not be performed routinely, because there is no evidence to suggest that this represents the best treatment. That said, the decision to perform one should take into account the personal physical and psychological burden resulting from local inflammation and the extent of fistula secretion in cases of management failure.

Interestingly, our study highlights that direct coloanal or colorectal anastomosis may be much more successful than all of the other procedures that can be performed. Their superiority is also suggested, albeit backed up with only little evidence, in the literature. Karakayali et al. [33], for instance, demonstrate the benefits of coloanal anastomosis for radio-induced rectovaginal fistulas, showing no recurrence at 20 months and a significant improvement in quality of life. Additionally, Woo et al. [34] achieved a success rate of 85.7% with coloanal anastomosis for the treatment of rectovaginal fistulas secondary to anterior rectal resection. Surprisingly, the delayed coloanal anastomosis was not superior to ERAF nor direct coloanal or colorectal anastomosis. This may be explained by the fact that, while it might have appeared promising at first, delayed coloanal anastomosis had a success rate of only 68% [35]. However, because of a specific morbidity superior to the other procedures, in the context of RVF, coloanal or colorectal anastomosis may be proposed in second intention, especially in complicated cases.

Our study has a number of limitations, primarily being a retrospective study with the known shortcomings of this study type. We lacked details on perioperative management conditions, as shown by Hiraki et al. [36], where certain perioperative elements can improve treatment outcomes. Additionally, information on fistula characteristics (size, location, etc.) was lacking, while Lohsiriwat [37] demonstrated that therapeutic results are influenced by several factors, including the size and location of the fistula. Data regarding the incidence of diabetes and smoking, both of which could influence the success rate of healing, were also not collected, nor was information on which examinations were performed prior to surgery. Our study does not address operative morbidity or functional outcomes, especially in terms of anal continence, while such information could be of interest in informing physicians which procedure to choose. Finally, the initial procedures leading to the RVF were heterogeneous leading to a probable bias in the interpretation of the analysis of the procedures or of the need for a stoma. However, despite this heterogeneity, this cohort is one of the most homogeneous because it included only RVF secondary to pelvic surgery, while the other cohort study that focused on this subject included all causes of RVF.

Despite these limitations, this study provides evidence that a diverting stoma is by no means necessary in postoperative RVF, because it does not improve the rate of success. Instead, our findings suggest that, despite mitigated results, ERAF may be performed as a first-line treatment. Not only is this because ERAF has a low risk of morbidity, but also because the only procedure that showed a greater success rate while direct coloanal or colorectal anastomosis, has a higher risk of morbidity.

## Conclusion

Our results suggest that creating a diverting stoma is not necessary in the management of postoperative RVF because it does not increase the success rate of the surgical procedures, nor of the overall management. An ERAF is effective in 1/3 cases and may be performed as first-line treatment intention before considering more morbid surgeries such as direct coloanal or colorectal.

## Data Availability

Data are available for reasonable request by addressing an e-mail to the corresponding author. No datasets were generated or analysed during the current study.
